# An update on non-thyroidal illness syndrome

**DOI:** 10.1007/s40618-020-01482-4

**Published:** 2020-12-15

**Authors:** E. Fliers, A. Boelen

**Affiliations:** 1 Department of Endocrinology and Metabolism, Amsterdam Gastroenterology Endocrinology and Metabolism, Amsterdam UMC, University of Amsterdam, Meibergdreef 9, 1105 AZ Amsterdam, The Netherlands; 2 Endocrine Laboratory, Department of Clinical Chemistry, Amsterdam Gastroenterology Endocrinology and Metabolism, Amsterdam UMC, University of Amsterdam, 1105 AZ Amsterdam, The Netherlands

**Keywords:** TH, Low T3 syndrome, Sick euthyroid syndrome, T3, rT3, T4, TRH, TSH, Deiodinase

## Abstract

The non-thyroidal illness syndrome (NTIS) was first reported in the 1970s as a remarkable ensemble of changes in serum TH (TH) concentrations occurring in probably any severe illness. Ever since, NTIS has remained an intriguing phenomenon not only because of the robustness of the decrease in serum triiodothyronine (T3), but also by its clear correlation with morbidity and mortality. In recent years, it has become clear that (parenteral) feeding in patients with critical illness should be taken into account as a major determinant not only of NTIS but also of clinical outcome. Moreover, both experimental animal and clinical studies have shown that tissue TH concentrations during NTIS do not necessarily reflect serum low TH concentrations and may decrease, remain unaltered, or even increase according to the organ and type of illness studied. These differential changes now have a solid basis in molecular studies on organ-specific TH transporters, receptors and deiodinases. Finally, the role of inflammatory pathways in these non-systemic changes has begun to be clarified. A fascinating role for TH metabolism in innate immune cells, including neutrophils and monocytes/macrophages, was reported in recent years, but there is no evidence at this early stage that this may be a determinant of susceptibility to infections. Although endocrinologists have been tempted to correct NTIS by TH supplementation, there is at present insufficient evidence that this is beneficial. Thus, there is a clear need for adequately powered randomized clinical trials (RCT) with clinically relevant endpoints to fill this knowledge gap.

## Introduction

Soon after the development of radioimmunoassays for thyroxine (T4), triiodothyronine (T3) and thyroid stimulating hormone (TSH) in the 1970s, a drop in serum thyroid hormone (TH) levels was reported both during starvation and illness. In mild illness, only a decrease in serum T3 levels was seen but with increasing severity and duration of the illness there appeared to be a drop in serum T4 as well. This decrease of serum TH levels was reported in a variety of species and pathologies including sepsis, myocardial infarction, and many more diseases. In contrast to the situation during primary hypothyroidism, however, decreased serum TH concentrations did not coincide with increased serum TSH. This typical ensemble of changes in serum TH concentrations was called the low T3 syndrome, the euthyroid sick syndrome (ESS) or the sick euthyroid syndrome (SES). A more appropriate designation, without presuming the metabolic status of the patient, is non-thyroidal illness syndrome, or NTIS. The focus in publications on NTIS has traditionally been on the change in serum TH concentrations in relation to particular pathologies. Early studies pointed to increased serum reverse T3 (rT3) resulting from decreased degradation of rT3 during NTIS, when several labs with access to immunoassays for both T3 and rT3 observed this discrepancy between these two metabolites of T4. However, it is now generally accepted that serum rT3 may be normal or even reduced in some NTIS cases. Once elevated, rT3 does not increase much more in patients with more severe illness.

This review will focus on more recent studies mainly centered around differential changes in TH metabolism depending on the organ and tissue studied. TH availability in these tissues during NTIS appears to be independent of decreased serum concentrations to a large extent. To illustrate our increasing understanding of the pathogenesis of these complex changes we will zoom in on effects of NTIS on organ-specific TH transporters, receptors and deiodinases. The role of inflammatory pathways in these non-systemic changes will be addressed, including recent studies on a novel role for TH metabolism in innate immune cells. Finally, clinical implications of NTIS, including the role of nutrition, will be briefly touched upon.

### TH transport and metabolism

In the past two decades it has become clear that TH needs to be taken up in cells by specific transporters including monocarboxylate transporter 8 (MCT8), monocarboxylate transporter 10 (MCT10) and organic anion transporting polypeptide family 1C1 (OATP1C1) to exert its action at the cellular level [1]. MCT8 and OATP1C1 are of crucial importance for the regulation of TH activity in the brain and thus for brain development [2]. In the past decade, effects of NTIS on TH transport has been studied in a variety of organs, including the liver (for review see Boelen et al. [3]). NTIS generally decreases TH transport in hepatocytes at the level of the plasma membrane. Using deiodination of T4 as a measure of cellular transport of T4 into rat hepatocytes, various older studies [4, 5, 6] reported that serum from patients with NTIS inhibits T4 uptake, mediated in part by increased non-esterified fatty acids (NEFA) and bilirubin and by decreased albumin. Interestingly, changes in liver TH transporter expression differs between various animal models of NTIS. For example, acute illness lowers *Slc16A2* (MCT8) and *Slc16A10* (MCT10) mRNA expression, whereas chronic inflammation results in a combination of decreased *Slc16A2* and increased *Slc16A10* mRNA expression. Unexpectedly, lethal bacterial sepsis affects hepatic TH transport only marginally [7]. In theory, reduced cellular TH uptake by itself would cause tissue hypothyroidism, but this is generally not observed. Therefore, it is likely that reduced cellular uptake, if present, is just one of many factors involved in NTIS.

Intracellular TH is metabolized via iodothyronine deiodinases, a group of selenium cysteine containing enzymes capable of removing an iodine atom from the inner or outer ring of TH. Iodothyronine deiodinases comprise type 1 deiodinase (DIO1) catalyzing inner- and outer-ring deiodination, type 2 deiodinase (DIO2) catalyzing only outer-ring deiodination (converting its preferred substrate T4 into T3), and type 3 deiodinase (DIO3) which catalyzes only inner-ring monodeiodination (converting its preferred substrate T3 into T2 but also T4 into rT3 thereby inactivating TH) [8]. Changes in DIO1, DIO2 and DIO3 expression and activity during NTIS have been reported in a variety or organs and tissues [3]. The ensemble of intracellular deiodinase activities determines the intracellular availability of T3, which may bind to TH receptors (TR) mediating both genomic (canonical) and non-canonical actions [9, 10]. Three T3 binding TRs are known, i.e., TRα1, TRβ1 and TRβ2. These TRs are important in regulating TH target genes in peripheral tissues and are differentially expressed between organs. Like the TH transporters and the deiodinases, TR expression levels are also affected in a differential way during NTIS [3]. Organ specific changes in TH transport, metabolism and action during NTIS will be discussed in some more detail in the next paragraph.

### Tissue and organ specific responses

A variety of changes in TH transporters, deiodinase and TR action have been observed in different organs and tissues both during acute and chronic illness, while serum TH levels are uniformly downregulated [3, 11]. Changes in TH metabolism in the hypothalamus, liver, muscle and innate immune cells will be described in more detail below.

### TH transporters

Hypothalamic MCT10 and OATP1C1 expression increases during critical illness in experimental animals while hypothalamic MCT8 mRNA expression does not change. Of note, these alterations are unrelated to local tissue T3 concentrations and their functional consequences are unknown at present [12].

TH transport in the human liver is differentially affected during illness: MCT8 expression increases in prolonged critical illness compared to acute illness, while MCT10 expression does not differ between these groups [13]. Likewise, acute and chronic NTIS animal models show differential changes in hepatic MCT8 and MCT10 expression. It is uncertain whether these alterations in liver TH transporter expression affect liver TH concentrations [7].

Like the liver, muscle is an important T3 target tissue and muscle TH metabolism is known to be affected during NTIS. In mice, muscle TH transporter expression is low during acute inflammation and bacterial sepsis but remains unchanged during chronic inflammation. Similar changes were observed in patients, as MCT8 expression was remarkably low in muscle tissue of patients during acute surgical stress compared to patients with prolonged illness [13]. Prolonged illness in rabbits results in markedly increased MCT10 expression while MCT8 mRNA expression does not change. In this case, serum TH may play a role as TH treatment of these rabbits restores muscle transporter expression to normal [13].

### Type 1, type 2 and type 3 deiodinases

The balance of deiodinase activities is an important determinant of TH availability in the cell. Deiodinase expression and activity are markedly affected during illness, depending on the severity and duration.

In the hypothalamus, DIO2 is expressed in glial cells and tanycytes and DIO3 is expressed in neurons of the paraventricular nucleus (PVN), supraoptic nucleus (SON) and IFN [14]. Illness results in increased DIO2 mRNA expression and activity [12, 15–17] while hypothalamic DIO3 decreases [18, 19]. By inference, hypothalamic TH availability probably increases during NTIS.

A healthy liver predominantly expresses DIO1, with very low DIO3 expression. Illness results in marked downregulation of DIO1 both in humans and experimental animals [15, 20, 21]. Liver DIO3 is differentially regulated during illness: acute inflammation in mice results in decreased DIO3 levels [15] but prolonged critical illness, both in patients and in rabbits, increases DIO3 [21, 22]. The response of DIO3 to illness might depend on energy status; fasting results in increased liver DIO3 activity in rodents [23, 24] and prolonged illness is associated with diminished food intake [25].

In muscle, local conversion of T4 toT3 by DIO2 is important for muscle function both in rodents and in humans [4, 26, 27]. Muscle DIO2 is differentially affected during illness. Acute inflammation in mice increases muscle *Dio2* mRNA expression, while bacterial sepsis decreases *Dio2* mRNA expression both in mice [28] and humans [29]. By contrast, DIO2 mRNA expression and activity remain unchanged in skeletal muscle tissue of patients with acute surgical stress compared to healthy controls [30]. Acute inflammation in mice decreases muscle DIO3 [31] while chronic inflammation in the same species increases muscle DIO3. Likewise, muscle DIO3 is increased in septic patients. The combination of differentially regulated DIO2 and DIO3 activities is likely to result in altered T3 bioavailability and, therefore, T3 signaling in muscle during illness. However, muscle UCP3 and myogenin mRNA expression, which are both T3 target genes, are unaltered in septic patients compared to controls [29]. The same observation was made in mice with chronic inflammation and bacterial sepsis [7]. This implies that decreased TR expression may be an adaptation to restore T3 signaling in muscle during sepsis.

Recently, it became clear that the cellular function of neutrophils and macrophages, both important players of the innate immune system, highly depends on the availability of intracellular TH, regulated via DIO3 and DIO2 respectively. DIO3 protein is highly expressed in infiltrating murine neutrophils during both bacterial infection and sterile inflammation [32, 33]. DIO3 is also expressed in human neutrophils [34] and present in intracellular granules involved in bacterial killing and in neutrophil extracellular traps (NETs) [34]. A lack of DIO3 impairs the bacterial killing capacity of mice upon infection with *Streptococcus pneumonia* [17] and clearly increases the mortality of zebrafish embryos with pneumococcal meningitis. Furthermore, NADPH-oxidase activity is impaired in stimulated neutrophils of DIO3 knock-out mice compared to wild type neutrophils [35]. These findings strongly support a critical role for the TH inactivating enzyme DIO3 in neutrophil function during infection. DIO2 is the main deiodinase present in macrophages and TRα1 is their dominant TR [36]. Activation of macrophages results in increased DIO2 expression together with increased TRα1 and MCT10, together pointing to increased TH action in these cells during inflammation [36]. In addition, DIO2 knockdown impairs macrophage phagocytosis while blunting the cytokine response upon stimulation with bacterial endotoxin [36, 37]. Finally, DIO2 knockdown increases mortality in zebrafish embryos during pneumococcal meningitis, which can be restored by T3 administration during infection [37]. Thus, increased intracellular T3 availability appears to play a crucial role in macrophage function.

### TH action

TH action is determined by intracellular T3 concentration and TR status. Both components have been studied during NTIS in both humans and experimental animals.

Hypothalamic TR mRNA expression does not change dramatically during NTIS [12], but the increase in hypothalamic DIO2 and decrease in hypothalamic DIO3 observed during acute inflammation is exaggerated in mice lacking TRβ [18]. This suggests that TRβ modulates the illness induced changes in hypothalamic deiodinase expression. T3 concentrations in post mortem human hypothalamic samples of NTIS patients are lower compared to patients with acute death from trauma [38].

Liver TRα and TRβ mRNA expression are differentially affected during illness, depending of its severity and duration. Liver TRα and TRβ expression in rodents decrease during acute illness [15, 39] but not during chronic inflammation and bacterial sepsis. In contrast, TRα and TRβ mRNA expression are increased in liver biopsies of patients with chronic liver disease prior to liver transplantation, in association with low serum T3 and T4 levels [40]. However, in patients with a variety of chronic liver diseases, hepatic TRα1, TRα2 and TRβ1 and T3-target gene expression are unchanged, indicating a euthyroid condition in the liver of these patients despite reduced serum TH levels [41]. These findings are in line with chronic inflammation mouse and rat models.

The effects of illness on T3 signaling in muscle also depends on the type of illness. UCP3, which is a T3 target gene, increases in skeletal muscle of mice during acute sepsis [42] and inflammation [43] as well as in skeletal muscle of NTIS patients, coinciding with increased DIO2 expression [44]. In contrast, chronic inflammation in mice results in simultaneously increased DIO2 and DIO3 activity [28]. This may result in a coordinated breakdown of both T4 and T3, finally leading to increased intracellular T2 levels. Of note, both T3 and T2 are known to regulate mitochondrial activity in skeletal muscle [45, 46]. TR expression in the diaphragm, the main respiratory muscle with high energy demands, is also affected during inflammation. Acute inflammation profoundly decreases TRα1 and TRβ1 mRNA expression in the mouse diaphragm in association with decreased expression of co-activators involved in nuclear receptor activation [47]. In the same animal model, bacterial sepsis reduces TH signaling coinciding with increased DIO3 and decreased DIO2 expression, while chronic inflammation decreases DIO3 expression [48].

The differential regulation of TH transporter, deiodinase and TR expression in acute inflammation, chronic inflammation and sepsis supports the concept that the net result of altered cellular TH metabolism during NTIS determines organ function during specific stages of illness.

### Mechanism of altered TH metabolism

Multiple mechanisms are known to be simultaneously involved in the pathogenesis of NTIS. One key element is the role of inflammation. Inflammatory mediators like cytokines and inflammatory signal transduction have been extensively studied in humans, experimental NTIS animal models and in vitro models [3]. An early clinical observation was that interleukin 6 (IL-6), a systemic, proinflammatory cytokine, is negatively correlated with serum T3 concentrations in hospitalized patients [49]. Other proinflammatory cytokines including interleukin 1β (IL-1β), soluble interleukin-2 receptor (sIL-2R) and tumor necrosis factor (TNF)α are elevated in septic NTIS patients [50]. In an acute NTIS mouse model, an increase in serum IL-6 and TNFα concentrations precedes the decrease in serum T3 and T4 [51]. A causal role for IL-6 is likely since IL-6 knockout mice show a smaller decrease in serum T3 during acute illness [52], although acute injection of IL-6 failed to induce NTIS in mice [51]. In support of an important role for IL-6, chronic IL-6 infusions result in decreased serum T4, T3 and hypothalamic TRH expression in rats [53]. In human volunteers, short-term infusion of IL-6 induces decreased serum TSH concentrations, but daily injections over 42 days only cause a modest decrease in T3 and a transient increase in rT3 and free T4 [54]. Like chronic IL-6 administration, chronic IL-1β administration in rats induces NTIS [55]. However, blockade of IL-1 signaling in healthy volunteers by recombinant human IL-1 receptor antagonist does not prevent the endotoxin induced alterations in THs [56]. The same holds true for TNFα, as infusion of this cytokine in humans alters serum T3, TSH and rT3, while blocking TNFα by a recombinant TNF receptor-IgG fusion protein does not prevent these changes [57]. Cellular studies clearly showed that proinflammatory cytokines can downregulate various components of TH synthesis and metabolism in thyrocytes [58] and hepatocytes [59–61]. In sum, many studies indicate a dominant role for cytokines in the pathogenesis of NTIS but fail to pinpoint one particular cytokine as the crucial mediator.

Recent studies indicate a role for the inflammatory signal transduction pathway nuclear factor κB (NF-κB) in the pathogenesis of NTIS. The NF-κB protein family consists of several subunits of which the 65kD subunit of NF-κB (RelA) activates the Dio2 promoter [16, 62]. Inhibition of RelA prevents the LPS-induced DIO2 increase in a cell culture of primary tanycytes [63], while tanycyte-specific knock down of RelA in mice showed that NF-κB is essential for the LPS-induced hypothalamic DIO2 increase and TRH decrease in vivo [64]. In addition to DIO2, NTIS affects D1 via NF-κB. This is clear, e.g., from the observation that the IL-1-β induced decrease in DIO1 and TRβ mRNA expression in HepG2 cells, which is a hepatocyte derived cell line, depends on NF-kB signaling [59, 60].

One of the key features of NTIS is the absence of increased serum TSH in the face of markedly decreased serum T3 and T4, pointing to altered set-point regulation of the hypothalamus-pituitary-thyroid (HPT) axis. One of the mechanisms involved is central downregulation of the HPT axis, resulting in persistently low serum TH concentrations. Indeed, reduced hypothalamic TRH expression, portal serum TRH and pituitary TSH content have been observed both in NTIS patients [65] and NTIS animal models [12, 19, 66, 67]. The illness-induced suppression of TRH in the hypothalamic paraventricular nucleus (PVN) is probably mediated by increased local T3 production, resulting from increased DIO2 expression in tanycytes in combination with decreased DIO3 expression in neurons [68]. Inflammatory mediators contribute to the illness induced alteration in both DIO2 and DIO3. Hypothalamic TRH is also modulated by glucocorticoids, as high glucocorticoid levels suppress the pituitary response to TRH in man [69], while a postmortem study in human hypothalamus showed that glucocorticoid treatment is associated with downregulation of TR neurons in the human PVN [14]. Furthermore, a stress-induced elevation of glucocorticoids in animals contributes to suppressed serum TSH, T4 and T3 levels [70]. Diminished food intake plays an additional role as NTIS is associated with diminished appetite and nutritional intake [71–74]. The drop in serum leptin during food deprivation is known to induce a decrease in TRH expression in the hypothalamic PVN via leptin receptor mediated changes in neuropeptidergic pathways, ultimately resulting in diminished TSH secretion from the anterior pituitary and decreased TH levels. Experimental studies in healthy volunteers during food deprivation showed that administration of leptin may partially reverse this process [75].

Besides inflammatory mediators, glucocorticoids and energy status, alternative mechanisms involving the peripheral metabolism of TH have been reported. Liver DIO1 expression and activity are downregulated during NTIS. The Dio1 gene is positively regulated via a TR/RXR heterodimer which suggests that decreased TR or/and reduced RXR expression may play a role in the downregulation of hepatic DIO1 during NTIS [76]. Indeed, liver TRβ1 expression decreases during acute inflammation in mice [15, 39, 77] and limited availability of SRC-1, a shared coactivator for TR and inflammatory signaling pathways, might be partly responsible for the illness induced decrease of liver DIO1 [78, 79]. In addition, decreased availability of glutathione (GSH), a co-factor required for DIO1 catalytic activity, may also play a role [80].

### Differential diagnosis and clinical implications

The clinical endocrinologist is frequently asked to provide endocrine consultation in case of a severely ill patient in the ICU without a (family) history of thyroid or pituitary disease, in whom a very low (F)T3 is found in combination with low or normal (F)T4 and low or normal TSH. It is clear that the typical clinical features of severe hypothyroidism with or without the presence of a goiter are absent in patients with NTIS, even in the presence of a very low serum T3. Thus, the combination of these features should lead to a high suspicion of NTIS. If both T3 and T4 are very low a potentially fatal outcome may be assumed. Potential caveats include a clearly elevated TSH suggesting the presence of prior hypothyroidism. There is no doubt that overt primary hypothyroidism in a patient with NTIS in the framework of severe illness should be treated with levothyroxine (LT4). Finding positive anti thyroid peroxidase (TPO) antibody titers supports the diagnosis of primary hypothyroidism but does not prove it. In addition to a high serum TSH concentration, a relatively high ratio of serum T3–T4, a low thyroid hormone-binding ratio and a low serum concentration of rT3 favor the presence of hypothyroidism over NTIS and vice versa. The determination of rT3 is, however, not routinely available in many hospitals and does not reliably distinguish between hypothyroidism and NTIS. As a potential complicating factor, serum TSH may be relatively low in critically ill patients with concomitant hypothyroidism as a result of a variety of drugs, for example dopamine which inhibits TSH release (for review see Fliers et al. [81]). Moreover, use of anticonvulsants and glucocorticoids should be noted, since these agents can lower (F)T4. Once a diagnosis of severe primary hypothyroidism in a critically ill patients with NTIS is established, many clinicians prefer a loading dose of 300–500 μg intravenous LT4 to quickly restore circulating levels of T4 to approximately 50% of the euthyroid value, followed by 50–100 μg of intravenous LT4 daily until oral medication can be given. If no clinical improvement is seen within 24 to 48 h after the onset of intravenous LT4 treatment, T3 may be considered (e.g., 10 μg given intravenously every 4 h or 25 μg given intravenously every 8 h) [82].

Hyperthyroidism is the typical cause of suppression of TSH below 0.01 mU/L, but it is rarely difficult to exclude this diagnosis in the setting of severely depressed T4 and T3.

The question whether interventions aimed at normalizing TH concentrations in patients with NTIS in the framework of protracted critical illness are beneficial has not been answered to date. Only a small number of RTCs involving heterogeneous NTIS patient groups, ranging from protracted critical illness to elective heart surgery in otherwise healthy participants, have assessed the effects of treatment with THs. Both T3 and levothyroxine have been used. The results of TH treatment in critical illness have been largely negative in terms of clinical benefit (for review, see Fliers et al.) [83]. This may be partly explained by the fact that NTIS is not limited to decreased serum TH concentrations during illness, but comprises complex organ and tissue-specific changes in TH metabolism as a reflection of marked changes in local inflammatory pathways, and nutritional status. Thus, normalizing TH concentrations in serum does not equal normalizing TH tissue concentrations and local TH action. This was clearly shown in a study involving patients who were critically ill and received TH treatment [84].

An unresolved issue is whether there is a place for TH treatment in patients with heart failure. Although the use of TH treatment in patients with heart failure has not been adequately studied, some trials have reported encouraging findings. A small RCT [85] in patients with dilated cardiomyopathy showed beneficial effects of medium-term (3 months) levothyroxine treatment on cardiac performance, whereas another RCT [86] showed that the TH analogue 3,5 di-iodothyropropionic acid improved some hemodynamic variables in heart failure.

An important question raised by dr Van den Berghe and colleagues is whether hypothalamic neuropeptides (e.g., growth hormone releasing hormone (GHRH), growth hormone releasing peptide 2 (GHRP2), gonadotropin releasing hormone (GRH), and TRH) can be used in patients with long-term critical illness in an attempt to stimulate the anterior pituitary gland, thereby restoring endocrine function in terms of plasma concentrations and hormone pulsatility [87]. A number of small studies showed that iv TRH in combination with GHRP2can restore circulating concentrations of TH and TSH pulsatility to a remarkable extent [88]. Moreover, this strategy improved bone markers and anabolic variables. These findings seem to point to insufficient hypothalamic drive of pituitary TSH release in protracted critical illness. This concept was supported by a study reporting that deceased patients with NTIS had decreased TRH mRNA expression in the hypothalamic paraventricular nucleus, correlating with decreased *ante mortem* plasma TSH and T3 concentrations [65]. However, at present there are no studies on the use of hypothalamic neuropeptides in patients with protracted critical illness reporting clinically relevant endpoints related to morbidity and mortality.

In sum, no definitive conclusion about the efficacy of TH treatment in patients in the ICU with NTIS can be made at present. The reported studies thus far were not adequately powered to detect clinically meaningful differences. Furthermore, most studies used rather high doses of either T4 or T3, probably inducing further suppression of pituitary TSH release. In this respect, the use of neuropeptides, including TRH, to stimulate the HPT axis may be more promising. Large RCTs in well-defined patient groups will be needed to assess possible positive effects of this approach in terms of clinical outcome. High priority should be given to RCTs comparing the effect of hypothalamic neuropeptides including TRH with placebo, because this approach has already been shown to partly normalize concentrations of serum THs and at the same time improve metabolic markers [89]. Another theoretical possibility would be to investigate treatment with recombinant human TSH, because this is a physiological stimulus—similar to TRH—for TH release from the thyroid.

## Remaining questions

### What is the relative role of reduced caloric intake?

Critical illness is associated with loss of appetite and poor nutritional intake. As fasting in healthy individuals induces complex changes in the HPT axis including low serum T3 without increased TSH, decreased caloric intake during severe illness is likely to contribute to the development of NTIS [90].

A large clinical trial reported comparisons between two nutritional regimens in ICU patients, i.e., an early parenteral nutrition group receiving parenteral nutrition within 48 h of admission to the ICU to supplement insufficient enteral nutrition, and a parenteral nutrition group who did not start this feeding regimen before day 8 in the ICU. Interestingly, the toleration of a nutritional deficit during the first week of critical illness resulted in fewer complications and accelerated recovery [91]. Ability to tolerate a fasting response thus seemed to be beneficial. Of note, a subanalysis of this trial showed that late feeding enhanced the changes in circulating concentrations of serum TH. Therefore, peripheral TH metabolism is clearly affected by decreased nutritional intake during acute critical illness. The subanalysis suggested that the inactivation of T3 to rT3, as part of the fasting response, might be a beneficial adaptation during acute illness [25]. In line, targeting of fasting blood glucose concentrations with intensive insulin treatment in children with critical illness to values mimicking a fasting response, resulted in improved outcomes, but at the same time aggravated peripheral NTIS. Further statistical analysis showed that the further reduction of T3 was associated with improving patient mortality rates [92, 93].

Together, these findings suggest that thyroid economy is affected by decreased nutritional intake during acute critical illness and that the inactivation of T4–rT3 and T3–T2, as part of the fasting response, might be a beneficial adaptation during acute illness. Teleologically, the reduced amount of circulating active T3 could be interpreted as an attempt by the body to decrease the metabolic rate, reduce energy expenditure, and prevent protein breakdown, thereby promoting survival. By contrast, the central lowering of T4 could be harmful (for review see Fliers et al., [83]. It should be noted, however, that at this time, there is no direct evidence support this hypothesis.

### What is the relative role of the hypothalamus vs. peripheral mechanisms in the fall of serum T3 and T3?

As pointed out in the previous paragraphs, both the central (hypothalamus and pituitary) and peripheral (thyroid gland and target organs) components of the HPT axis show robust changes during NTIS. Studies in mice on the time course of the changes in thyroid hormone metabolism in the HPT axis during acute illness induced by bacterial endotoxin (lipopolysaccharide; LPS) showed a rapid induction of interleukin-1beta mRNA expression in the hypothalamus, pituitary, thyroid and liver. This was followed by almost simultaneous molecular changes in the pituitary, the thyroid gland and the liver. In the hypothalamus, D2 mRNA was strongly increased whereas preproTRH mRNA expression did not change after LPS. Serum T3 and T4 fell only after 24 h. Together, this suggested almost simultaneous involvement of the whole HPT axis in the downregulation of thyroid hormone metabolism during acute illness [15].

In the clinical setting, less information is available on the timing and the relative role of central versus peripheral mechanisms in the pathogenesis of NTIS. Peripheral mechanisms related to macronutrient restriction occur already in the acute phase in critical illness and appear beneficial. In the prolonged phase of critical illness, hypothalamic thyrotropin releasing hormone (TRH) expression is suppressed as shown both in animal and human studies, contributing to reduced TSH secretion and reduced thyroidal hormone release. The clinical implications of NTIS in the prolonged phase of critical illness when patients receive nutrition but continue to depend on intensive medical care, is uncertain.

Many experimental and clinical studies pointed to a biphasic neuroendocrine pattern in critical illness as conceptualized by dr Van den Berghe and colleagues (see reviews [87, 94]). Indeed, many studies support the concept that the acute phase of critical illness induces rapid neuroendocrine changes directing the organism toward a catabolic state to ensure provision of energy sources, postponing anabolic processes. However, in many patients admitted to the ICU a subsequent prolonged phase of critical illness sets in. This prolonged phase of critical illness is characterized by an overall suppression of several neuroendocrine axes including the HPT axis. No solid data are available to answer the question if the neuroendocrine changes in prolonged critical illness are harmful and should be treated.

### Is thyroid hormone signaling disrupted during prolonged NTIS?

During prolonged critical illness, several tissue responses reported from both animal and human studies could be interpreted as compensatory to low thyroid hormone availability. These responses include increased tissue expression of monocarboxylate transporters, upregulation of type-2 deiodinase activity, and increased sensitivity at the receptor level [87]. Infusing hypothalamic releasing factors, including TRH, in these prolonged critically ill patients has been shown to be able to reactivate the thyroid axis and induce an anabolic response. Unfortunately at this stage we do not know whether this approach can improve clinical outcomes. These investigations should be done in adequately powered, randomized, placebo-controlled studies.

## Conclusion

**Fig. 1:**
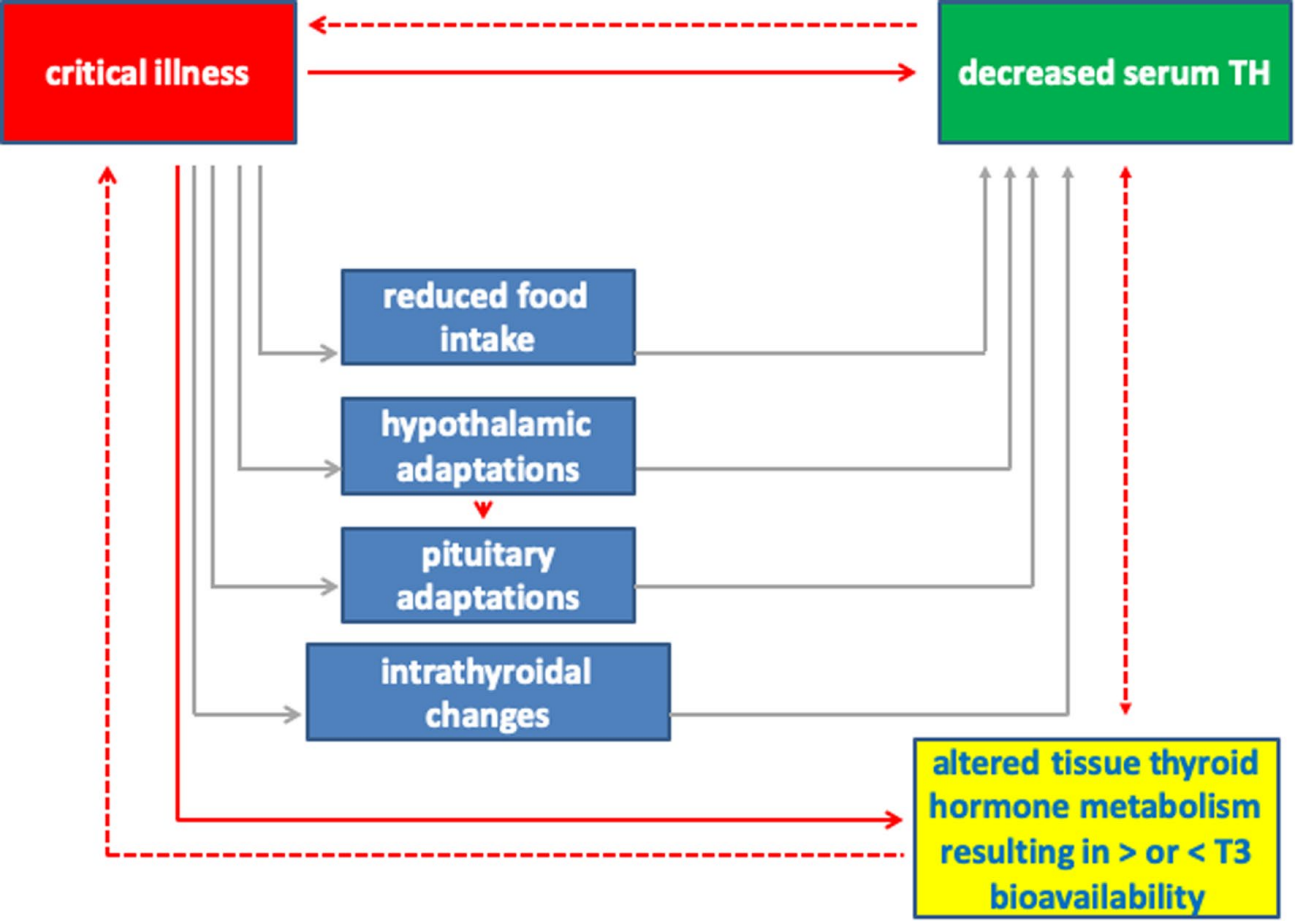
Schematic representation of the variety of changes occurring during critical illness. Solid lines represent a causal relation, while a dashed line represents a probable effect. The scheme is based on both experimental and human studies. The net result of altered tissue TH metabolism may be beneficial or maladaptive, dependent on disease duration and severity. Reproduced from Fliers E et al. Lancet Diabetes Endocrinol 3: 816–825, 2015 [83] with permission from Elsevier

NTIS has traditionally been seen as a syndrome defined by lower plasma TH concentrations of unknown significance. However, more recent studies have clearly shown that NTIS rather represents a complex set of marked and differential changes in TH physiology, both at the level of the HPT axis and at the organ and tissue level (Fig. 1).

Whether the observed changes in critically ill patients defined as NTIS are beneficial or deleterious is currently unknown. This may depend on disease type and severity, the need of prolonged vital support, and environmental factors including parenteral nutrition. At present, there is no clinical evidence to support TH treatment of NTIS in critically ill patients. There is a clear need to perform adequately powered RCTs to define whether pharmacological management of NTIS, e.g., using neuropeptides including TRH, may yield benefit in terms of clinical outcome.
